# Advances in cloning functional genes for rice yield traits and molecular design breeding in China

**DOI:** 10.3389/fpls.2023.1206165

**Published:** 2023-06-19

**Authors:** Qianqian Zhong, Qiwei Jia, Wenjing Yin, Yuexing Wang, Yuchun Rao, Yijian Mao

**Affiliations:** ^1^ College of Life Sciences, Zhejiang Normal University, Jinhua, Zhejiang, China; ^2^ State Key Laboratory of Rice Biology and Breeding, China National Rice Research Institute, Hangzhou, China

**Keywords:** rice, genes, yield traits, molecular markers, molecular design breeding

## Abstract

Rice, a major food crop in China, contributes significantly to international food stability. Advances in rice genome sequencing, bioinformatics, and transgenic techniques have catalyzed Chinese researchers’ discovery of novel genes that control rice yield. These breakthroughs in research also encompass the analysis of genetic regulatory networks and the establishment of a new framework for molecular design breeding, leading to numerous transformative findings in this field. In this review, some breakthroughs in rice yield traits and a series of achievements in molecular design breeding in China in recent years are presented; the identification and cloning of functional genes related to yield traits and the development of molecular markers of rice functional genes are summarized, with the intention of playing a reference role in the following molecular design breeding work and how to further improve rice yield.

## Introduction

With the development of urbanization and population growth, people have new demands for material living standards. As one of the main food crops for human survival, rice has new requirements in production, quality, and plant breeding (Zeng et al., 2017a). In modern breeding history, the effective utilization of dwarfing and heterosis resulted in two dramatic leaps in crop yield per unit area. Since the beginning of the 21^st^ century, China’s rice production has faced serious challenges. As the world population’s need for higher grain yield and superior quality escalates, building upon our current successes to further enhance rice yield becomes essential. This endeavor necessitates a comprehensive analysis of the intricate regulatory networks governing genes associated with major yield traits in rice. Utilization of key yield-related genes, or quantitative trait loci (QTLs), coupled with the discovery of additional genes with the potential to increase rice yield, is crucial. By doing so, we can devise innovative breeding strategies that circumvent traditional breeding limitations, propelling our efforts toward meeting the escalating demand. The application of molecular marker-assisted selection in rice breeding has been conducted for many years in China, leading to the construction of multiple chromosome segment substitution lines (CSSLs) and the identification of many QTLs. With the completion of rice genome sequencing and the mapping and isolation of important yield genes, significant progress has also been made in the functional analysis of these genes, providing the foundation for the application of molecular design in rice breeding (Zhou Y. et al., 2017).

In the practice of genetic improvement of crop yield-related traits, the breakthrough in yield potential is to make new progress in plant type while ensuring a high grain weight. Specifically, crop yield can be increased by improving panicle morphology, tiller traits, leaf shape, and angle, in addition to increasing the field planting density of plants to improve the light energy utilization rate, thus changing the number of grains per panicle and 1000-grain weight to increase the crop yield. Therefore, the development of plant architecture and grain formation are key traits for current and future high-yield breeding, and the analysis of genetic regulatory networks provides an important basis for improving these traits. In the case of rice, the analysis of rice plant architecture development (e.g., tiller number, plant height, tiller angle, leaf shape, stem and leaf angle, and panicle type) and grain formation (e.g., flower/panicle completion and grain development), which affect rice yield and the molecular genetics, physiological, and biochemical regulatory networks of important biological processes closely related to them, proposes the molecular design and breeding theory of major rice yield traits, which can provide support for the cultivation of high-yield varieties of rice in China. Chinese scientists have made a series of achievements in the cultivation of high-yield and high-quality varieties of rice. In particular, the research achievements of Jiayang Li, Bin Han, and Qian Qian’s team, “Molecular mechanism and variety design of the formation of high-yield and high-quality traits of rice” won the first prize of the State Natural Science Award in 2017 - another breakthrough after the “green revolution” and “hybrid rice” in China, which is of great significance to the promotion of the development of molecular design breeding in China (Chen F. et al., 2018).

The current study classifies and summarizes the gene cloning and molecular design breeding of various important yield traits of rice in China in recent years and provides a prospective investigation on the future direction of molecular breeding of rice. Given the numerous accomplishments of Chinese researchers in rice genomics, there may have been unintentional oversights in compiling this review. This review of genes and gene regulatory networks is summarized as an example.

## Research advances in rice yield traits

**Figure 1:**
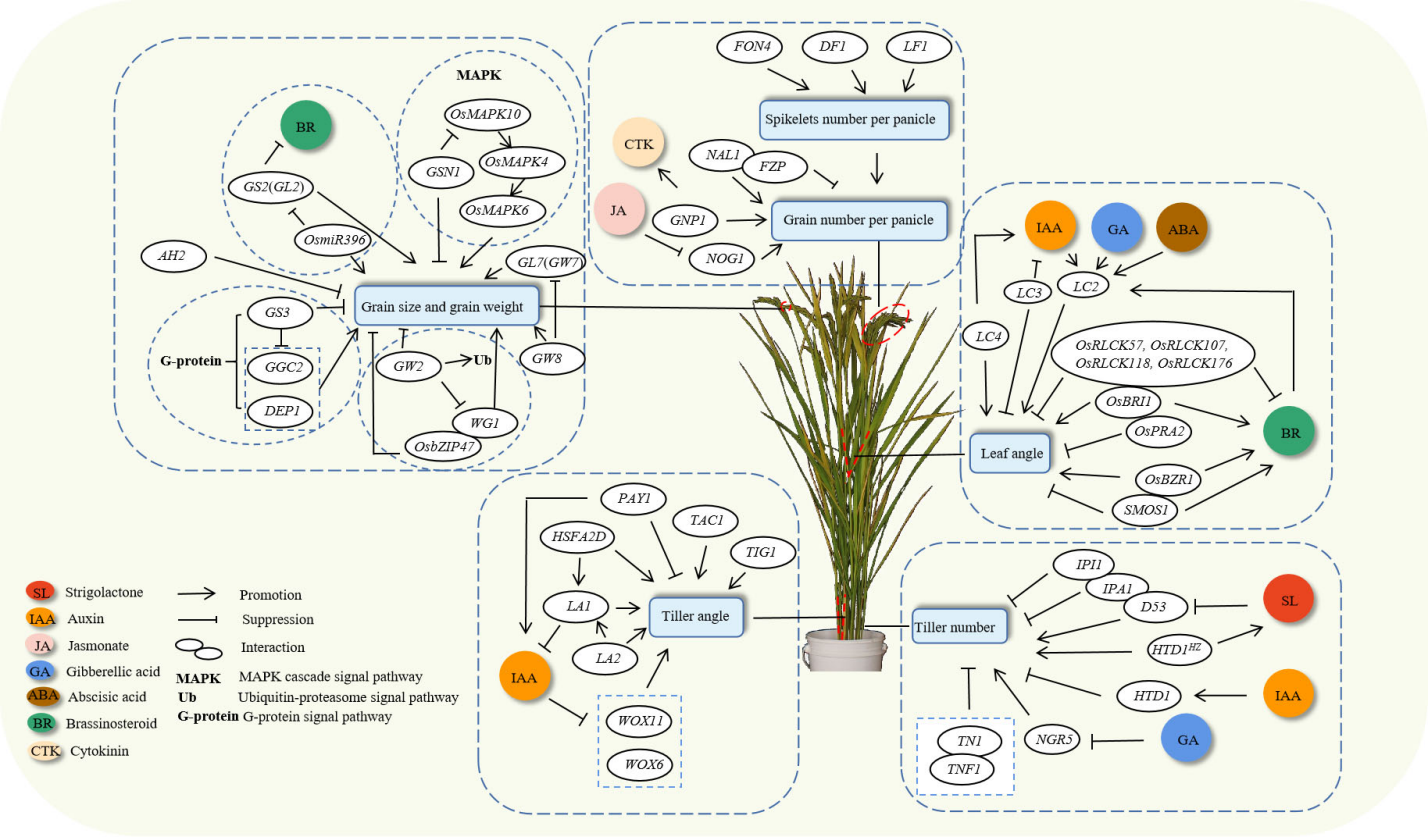
Gene regulatory network for yield traits.

The complex quantitative traits that determine rice yield are governed by numerous genes and highly sensitive to environmental variables. The key determinants of rice yield encompass plant morphology, panicle structure, and grain type. These yield characteristics mutually supplement each other to collectively determine the overall yield. Research teams in China have discovered and cloned a series of genes associated with these yield traits and shed light on the gene regulatory networks that influence these traits (**Figure 1**), substantially advancing the progress of rice research in the country.

## Cloning and genetic network analysis of key rice plant type genes

Rice plant type traits mainly include tiller number, tiller angle, and angle between stem and leaf, which are important agronomic traits that determine rice yield. Chinese breeders have carried out extensive research on the idealization of rice plant type, cloned a series of key genes that regulate plant type (**Table 1**), constructed relevant genetic regulatory networks, and cultivated many super rice varieties with ideal plant type characteristics.

**Table 1:** Representative functional genes for the characteristics of rice plants.

Trait	Gene	Coding product	Regulatory phenotype	Reference
Plant type	*IPA1*	transcription factor OsSPL14	ideotype	(Jiao et al., 2010)
	*IPI1*	RING-finger E3 ligase	tiller number and panicle size	(Wang and Wang, 2017)
	*NGR5*	AP2-domain transcription factor	tiller number	(Wu et al., 2020)
	*TN1*	functional protein with a BAH domain and an RNA recognition domain	tiller number	(Zhang et al., 2023)
	*D53*	a substrate of the SCFD3 ubiquitination complex	tiller number	(Jiang et al., 2013; Zhou et al., 2013)
	*HTD1*	carotenoid cleavage dioxygenase 7	tiller number	(Zou et al., 2006)
	*HTD1^HZ^*	carotenoid cleavage dioxygenase 7	tiller number	(Wang et al., 2020)
	*SD1*	gibberellin 20-oxidase	plant height	(Wang et al., 2020)
	*TAC1*	specific proteins of graminaceous plants	tiller angle	(Yu et al., 2007)
	*TIG1*	TCP transcription factor	tiller angle	(Zhang et al., 2019)
	*LA2*	a novel chloroplastic protein	tiller angle	(Huang et al., 2021)
	*LA1*	a novel and unique protein	tiller angle	(Li et al., 2007)
	*HSFA2D*	heat shock transcription factor	tiller angle	(Zhang et al., 2018)
	*WOX6*	WUSCHEL-related homeobox domain-containing protein	tiller angle	
	*WOX11*	WUSCHEL-related homeobox domain-containing protein	tiller angle	
	*PAY1*	containing a peptidase S64 domain	tiller number and tiller angle	(Zhao et al., 2015)
	*OsBZR1*	BR-signaling factor	leaf angle	(Qiao et al., 2017)
	*SMOS1*	AP2-domain transcription factor	leaf angle	
	*OsPRA2*	small GTP-binding protein	leaf angle	(Zhang et al., 2016)
	*OsBRI1*	BR receptor kinase	leaf angle	
	*OsRLCK107*	receptor-like cytoplasmic kinase	leaf angle	(Zhou et al., 2016)
	*OsRLCK57*	receptor-like cytoplasmic kinase	leaf angle	
	*OsRLCK118*	receptor-like cytoplasmic kinase	leaf angle	
	*OsRLCK176*	receptor-like cytoplasmic kinase	leaf angle	
	*LC3*	a SPOC domain-containing transcription suppressor	leaf angle	(Chen SH. et al., 2018)
	*LC4*	F-box protein	leaf angle	(Qu et al., 2019)
	*LC2*	a vernalization-insensitive 3-like protein	leaf angle	(Zhao et al., 2010)

### Regulatory network for rice tiller numbers

The quantity of tillers in rice plants is a primary factor influencing yield. Moreover, it is a crucial agronomic attribute that determines tolerance to density and resistance to lodging. It has been demonstrated that the gene *Ideal Plant Architecture1* (*IPA1*), one of the major rice genes responsible for ideal plant architecture, encodes a transcription factor called OsSPL14 (SOUAMOSA PROMOTER BINDING PROTEIN-LIKE 14), which is involved in the regulation of multiple growth and developmental processes (Jiao et al., 2010). In-depth research on the interaction protein system of the IPA1 protein revealed that it associates with IPA1 INTERACTING PROTEIN1 (IPI1), a nuclear-localized RING-finger E3 ligase that can ubiquitinate the IPA1 protein. Haiyang Wang, affiliated with the Chinese Academy of Agricultural Sciences, expressed his thoughts on this gene in the journal Molecular Plant. He indicated, “The loss of *IPI1* function can increase the number of tillers and the size of panicles, which in turn can increase yield. This gene is expected to be applied in variety modification to increase yield by increasing tiller number” (Wang and Wang, 2017). In an article recently published in Cell Research, Reynante et al. argued that *IPI1* is a candidate gene with yield-increasing potential that can improve panicle number without sacrificing the number of tillers (Ordonio and Matsuoka, 2017). Further biochemical analysis has revealed that the ubiquitination of IPA1 protein by IPI1 protein is tissue-specific, allowing fine regulation of IPA1 protein levels in different tissues. The natural allelic combination of different *IPI1* genes results in different levels of *IPA1* expression, which affect the number of tillers in a dose-dependent manner and allow for achieving a reasonably balanced number of tillers and the highest yield potential (Wang et al., 2017). Thus, rice breeders can create plant architecture characteristics that meet specific farming environments and genetic backgrounds to improve the controllability of breeding. In addition, Xiangdong Fu’s group screened an EMS mutant library with a background of the semidwarf cultivar 9311 and identified a nitrogen-insensitive gene, *Nitrogen-mediated Tiller Growth Response5* (*NGR5*). This led to significant advances in the understanding of the gibberellin (GA) response to nitrogen in rice. Research has found that *NGR5* positively regulates rice growth and development in response to nitrogen (agronomic traits such as plant height, tillering, and grains per panicle) and can negatively regulate the expression of tillering inhibitory genes, thereby promoting tillering in rice. Further research has found that the NGR5 protein can interact with GIBBERELLIN INSENSITIVE DWARF1 (GID1), which inhibits the growth of rice tillering by promoting the degradation of the NGR5 protein (Wu et al., 2020). This study elucidated the key molecular mechanism of the intersection of the GA signaling pathway and nitrogen allocation and utilization. This finding provides a new target for advancing the high yield and efficiency goals of the “Green Revolution”. Professor Zichao Li from the College of Agriculture of China Agricultural University recently cloned a new rice tillering regulatory gene, *Tiller Number1* (*TN1*), which encodes a functional protein containing the BAH and RNA recognition domains. It negatively regulates the establishment of rice tillering numbers. At the same time, the team also found that the natural variation in the TN1 protein would affect its interaction with TN1 INTERACTION FACTOR1 (TIF1). When the *TIF1* gene was knocked out, the number of plant tillers increased. Therefore, *TN1* and *TNF1* have similar regulatory effects on the number of tillers in rice (Zhang et al., 2023). This research provides excellent gene resources and genetic information for designing the ideal type of rice plant. As a new type of hormone, strigolactone (SL) plays a key role in regulating the number of tillers in rice. Jiayang Li’s research group and Jianmin Wan’s research group published articles on the regulation of SL in rice tiller number in the journal Nature, revealing that *Dwarf 53* (*D53*) is a negative regulatory factor in the SL signaling pathway and that SL can induce *D53* degradation through the proteasome pathway, suppress lateral bud growth, and regulate the number of tillers in rice (Jiang et al., 2013; Zhou et al., 2013). In addition, Song et al. found that *D53* interacts with the ideal plant type gene *IPA1*, jointly regulating the number of rice tillers (Song et al., 2017). Recently, there has been a new development in the study of SL regulation in the number of rice tillers. In the research by Zou et al., the *TilleringI and Dwarf 1* (*HTD1*) gene, which encodes CAROTENOID CLEAVAGE DIOXYGENASE 7 (CCD7), negatively regulates the number of rice tillers, and IAA can upregulate the transcription of this gene to control them (Zou et al., 2006). Further research by Wang et al. has demonstrated a partial loss-of-function allele of the SL biosynthesis gene, *HTD1^HZ^*, which can increase the number and yield of rice tillers. At present, this gene and the semidwarf gene *Semidwarf 1* (*SD1*) are jointly selected to be widely used, which has to some extent contributed to the green revolution in rice (Wang et al., 2020).

### Regulatory network for rice tiller angles

An appropriate tiller angle is critical for the growth of rice and the formation of farmland yield. Recently, several genes controlling rice tiller angle have been identified, and their function in the network regulating rice plant architecture has been analyzed to some extent. *Tiller Angle Control 1 (TAC1)* is an important QTL previously reported for rice tillering angle regulation (Yu et al., 2007). The laboratory of Professor Chuanqing Sun at the China Agricultural University isolated a gene, *TAC1*, corresponding to the main QTL that controls rice tillering angle through positional cloning. The analysis of the plant architecture of *TAC1*-overexpressing and RNAi transgenic lines indicated that the upregulation of *TAC1* resulted in an increased tiller angle, while its downregulation decreased the tillering angle. These findings document that the level of *TAC1* mRNA determines the size of the tillering angle (Jiang et al., 2012). Another research team identified a Tiller Inclined Growth1 (*TIG1*) gene that affects tillering angle in wild rice and positively regulates cell elongation and tillering angle size. It encodes the transcription factors TEOSINTE BRANCHED1, CYCLOIDEA AND PCF (TCP) and is expressed on the paraxial side of the tillering base. After the artificial selection of the indica rice variety *tig1*, the natural variation of its promoter caused a significant decrease in the expression of the *TIG1* gene, resulting in a decrease in cell length and tillering angle (Zhang et al., 2019). This achieved the transition of wild rice from inclined growth to vertical tillering growth and provided strong support for the design of the ideal rice plant type.

Recently, a research group used map-based cloning to identify a gene, known as *LAZY2* (*LA2*), that controls tiller angle. This gene encodes a novel chloroplast protein that specifically governs starch in gravity-sensitive cells. By interacting with the starch biosynthetic enzyme, *Oryza sativa* plastidic phosphoglucomutase (OspPGM), *LA2* regulates the tillering angle, indicating the importance of gravity response in shaping the tiller angle (Huang et al., 2021). Additionally, a group of researchers obtained dynamic transcriptome data by combining RNA-seq technology to study the dynamic development process of rice stem gravity response and excavated important nodal genes that regulate the formation of rice tiller angles, such as *Heat Stress Transcription Factor2D* (*HSFA2D*), *LAZY1* (*LA1*), *Wuschel Related HomeoboX6* (*WOX6*), and *Wuschel Related HomeoboX11* (*WOX11*), and constructed a molecular network for the dynamic regulation of rice tillering angle at the whole genome level. Among them, *LA1* is a previously identified classical tiller angle-regulating gene. Previous studies have confirmed that *LA1* controls the gravitropism of the aboveground portion of the rice shoot and ultimately controls the size of the tillering angle by regulating the polar transport of auxin (IAA) (Li et al., 2007). Genetic evidence confirmed that *LA1* and SL may co-participate in regulating aboveground gravitropism and tillering angle in the auxin transport pathway (Sang et al., 2014). *HSFA2D*, *WOX6*, and *WOX11* are new regulatory genes implicated in the regulatory pathway mediated by *LA1. HSFA2D* is located upstream of the *LA1*-mediated auxin pathway and is a positive regulator of this pathway, implicating that *HSFA2D* can cause an unbalanced distribution of auxin by inducing the expression of *LA1*. Auxin promotes the asymmetric expression of the *WOX6* and *WOX11* genes, producing changes in the tiller angle (Zhang et al., 2018). Other genetic analyses show that the *LA2* gene also causes the change in tillering angle by acting upstream of *LA1* to mediate the lateral transport of auxin (Huang et al., 2021). In addition, Zhao et al. have made important progress in the research direction of polar transport of auxin to control the tillering angle of rice using map-based cloning technology. A novel gene, *Plant Architecture and Yield1* (*PAY1*), which controls plant architecture and yield, was cloned, and it was found that its mutation alters the absorption and polar transport capacity of auxin, and then changes the polar distribution of auxin *in vivo*, which is an important reason for the change in tillering angle of *PAY1*. *PAY1* is pleiotropic, and its overexpression enables smaller tillering angles, a lower number of tillers, increased plant height, thicker stalks, increased numbers of panicles, and higher yield (Zhao et al., 2015). This provides new ideas for research in this field by exploring an effective way to mine regulatory genes and regulatory pathways of the rice tiller angle.

### Regulatory network for stem-leaf angle in rice

Leaf angle affects the light-receiving posture of plants, and the appropriate angle is beneficial to increase the light-receiving area of the leaves and improve the light energy utilization rate. Presently, the genes regulating leaf angle are mostly related to the synthesis and signaling pathways of brassinolide (BR) and IAA. *Oryza sativa Brassinazole-resistant1* (*OsBZR1*), which encodes the BR-signaling factor, interacts with *Small Organ Size1* (*SMOS1*) to enhance its transcriptional activity. *SMOS1* encodes a transcription factor with an APETALA2 (AP2) DNA-binding domain, reduces the angle between rice stem and leaf, and plays an important role in BR signaling in rice (Qiao et al., 2017). *Oryza sativa Brassinosteroid Insensitive1* (*OsBRI1*) is the orthologue of *Arabidopsis thaliana Brassinosteroid Insensitive1* (*AtBRI1*) (Yamamuro et al., 2000). Several genes have recently been reported to interact with it, including *Oryza sativa Pea Pra2* (*OsPRA2*), *Oryza sativa receptor-like cytoplasmic kinase107* (*OsRLCK107*), *Oryza sativa receptor-like cytoplasmic kinase57* (*OsRLCK57*), *Oryza sativa receptor-like cytoplasmic kinase118* (*OsRLCK118*), and *Oryza sativa receptor-like cytoplasmic kinase176* (*OsRLCK176*). *OsPRA2* encodes a small GTP-binding protein, and its overexpression is characterized by a smaller leaf inclination and reduced sensitivity to exogenous brassinolide (Zhang et al., 2016). *OsRLCK57*, *OsRLCK107*, *OsRLCK118*, *OsRLCK176*, and other *OsRLCKs* encode receptor-like cytoplasmic kinases that interact with the BR receptor *OsBRI1* to suppress BR signaling and affect leaf inclination (Zhou et al., 2016). Chen et al. showed that the *Leaf Inclination 3* (*LC3*) gene, which controls the angle between the stem and leaf, encodes a transcriptional suppressor containing a SPOC domain and plays a role in suppressing IAA homeostasis and signal transduction. In addition to its involvement in regulating the stem-leaf angle mechanism, it also ensures the normal development of lamina joints (Chen SH. et al., 2018). Qu et al. found that microRNAs also play a certain role in regulating the angle between stems and leaves. This study shows that miR394 regulates the angle between stem and leaf by targeting the gene *Leaf Inclination 4* (*LC4*), and IAA will affect the transcription of miR394 and *LC4 (* Qu et al., 2019). Other plant hormones such as GA, jasmonic acid (JA), abscisic acid (ABA), and ethylene (ETH) also participate in the regulation of the angle between rice stems and leaves (Li QF. et al., 2019), and all of them achieve the regulatory function by affecting the synthesis and signal transmission of BR (Gan et al., 2015). Zhao et al. obtained a *Leaf Inclination 2* (*LC2*) gene that positively regulates the angle between stem and leaf through positional cloning and encodes a vernalization-insensitive 3-like protein. This gene is mainly expressed at the lamina joint, and its expression is influenced by GA, ABA, IAA, and BR (Zhao et al., 2010).

## Cloning and genetic network analysis of key genes for rice panicle type

Panicle type is a key factor in rice yield. Genetic analysis shows that it is controlled by multiple genes, mainly by influencing the number of grains per panicle and panicle morphology (the number of spikelets) to affect rice yield. In recent years, scientists have made great progress in exploring panicle type genes (**Table 2**) and have analyzed the regulatory network of rice panicle type to some extent.

**Table 2:** The representative functional genes for rice panicle type traits.

Trait	Gene	Coding product	Regulatory phenotype	Reference
Panicle type	*GNP1*	GA 20-oxidase	grain number per panicle	(Wu et al., 2016)
	*NOG1*	enoyl-CoA hydratase	grain number per panicle	(Huo et al., 2017)
	*FZP*	ERF transcription factor	grain number per panicle and 1000 grain weight	(Bai et al., 2017)
	*NAL1*	trypsin-like serine protease	grain number per panicle	(Huang et al., 2018)
	*LF1*	HD-ZIP III transcription factor	spikelet number per panicle	(Zhang et al., 2017)
	*DF1*	lipase	spikelet number per panicle	(Ren et al., 2017)
	*FON4*	small secreted protein containing the CLE domain	spikelet number per panicle	(Ren et al., 2019b)

### Regulatory network for rice grain number per panicle

The number of grains per panicle is important in determining rice yield. Wu et al. reported that the *Grain Number per Panicle1* (*GNP1*) gene, which encodes GA 20-oxidase, can increase grain number and yield by upregulating cytokinin (CTK) activity in rice panicle meristems (Wu et al., 2016). Since then, researchers have identified a gene, *Number Of Grains1* (*NOG1*), that affects the number of grains per panicle and rice yield. This gene is located on the long arm of chromosome 1 and encodes enoyl-CoA hydratase of the fatty acid β-oxidation pathway. A 12-bp transcription factor binding site in the *NOG1* promoter region exhibits copy number variation, which can increase the gene expression level, decrease the concentration of fatty acids and JA in the plant, and increase the number of panicle grains and yield. Introducing *NOG1* into the *NOG1-*deficient cultivar “Zhonghua 17” increases the yield by 25.8%, and overexpression of *NOG1* in the high-yielding cultivar “Teqing” carrying this gene increases the yield by nearly 20% without affecting plant height, heading date, panicle number, grain weight, and other traits. The cloning of *NOG1* provides an important new gene-based strategy for breeding high-yielding rice varieties and offers new insights into the molecular mechanisms underlying the regulation of traits that determine rice yield (Huo et al., 2017). Recently, it has been documented that rice yield is controlled by modulating the expression level of *Frizzy Panicle* (*FZP*). The manipulation of the *FZP* gene in rice leads to a surge in the number of grains per panicle, but with smaller grain sizes, whereas its overexpression triggers the growth of larger grains but decreases the number of grains per panicle (Bai et al., 2017; Ren DY. et al., 2018). The expression level of *FZP* determines the number of grains per panicle and the 1000-grain weight. Fine-tuning the balance between these two parameters to maximize yield by modulating *FZP* expression warrants further exploration. Further functional analysis showed that the trypsin-like serine protease encoded by *Narrow Leaf 1* (*NAL1*) interacts with the FZP protein, promoting its degradation. Reducing the expression of *FZP* or elevating the expression of *NAL1* in the cultivar Zhonghua 17 increases the number of secondary branches and grains per panicle, leading to a higher rice grain yield (Huang et al., 2018).

### Regulatory network for rice spikelet number per panicle

Another critical factor influencing the formation of grains per panicle that is often overlooked is the number of spikelets in a panicle. A standard rice panicle comprises two pairs of glumes and one fertile floret, which directly contributes to grain yield. Zhang et al. reported a rice mutant, *lateral florets1* (*lf1*). The spikelet of this mutant produces normal terminal florets, while the lateral floret meristem at the protective glume develops into relatively normal lateral florets. The *LF1* gene is the first gene identified to regulate the growth of lateral florets in rice and encodes the HD-ZIPIII protein; its mutation induces ectopic expression of the meristem maintenance gene (Zhang et al., 2017). This study provides strong evidence for the “three flowered spikelets” hypothesis in rice. Ren et al. identified a rice mutant called *double floret1* (*df1*) that develops an intact floret and produces normal seeds within a pair of nursing spikelets and the normal terminal florets. The *DF1* gene encodes a lipase that regulates the determinacy of the spikelet meristem, and its mutation changes spikelet certainty to uncertainty or prolongs the spikelet determination phase, resulting in the formation of multiple grains (Ren et al., 2017). These findings provide an important basis for research aimed at increasing the number of grains per panicle in rice. In a recent study, Ren et al. discovered an important gene *Floral Organ Number4* (*FON4*), which is involved in inflorescence and floral organ morphogenesis in rice. They identified a novel allelic mutant, *fon4-7*, which forms a lateral floret in addition to normal terminal florets, but different *fon4* allelic mutants have different numbers of florets (Ren et al., 2019b). This study shows that mutations in *FON4* provide the potential to form multiflowered spikelets, which in turn form multiple seeds.

## Cloning and genetic network analysis of key genes for rice grain types

Rice grain type is an important trait that determines grain weight and affects rice yield and quality. To date, researchers have cloned a series of genes related to grain type (**Table 3**), revealing that these genes regulate multiple signaling pathways and have made some progress in balancing rice yield and quality.

**Table 3:** The representative functional genes for rice grain type traits.

Trait	Gene	Coding product	Regulatory phenotype	Reference
Grain type	*GS2/GL2*	growth-regulating factor	grain size and length	(Che et al., 2015; Hu et al., 2015)
	*OsmiR396*	microRNA	grain size	(Duan et al., 2015)
	*GS3*	heterotrimeric G-protein γ subunit	grain length	(Sun et al., 2018)
	*GGC2*	heterotrimeric G-protein γ subunit	grain length	
	*DEP1*	heterotrimeric G-protein γ subunit	grain length	
	*GW2*	RING-type E3 ubiquitin ligase	grain width and weight	(Song et al., 2007)
	*WG1*	glutathione	grain size	(Hao et al., 2021)
	*OsbZIP47*	bZIP transcription factor	grain size	
	*GSN1*	mitogen-activated protein kinase phosphatase	grain size	(Guo et al., 2018)
	*GW7/GL7*	*Arabidopsis thaliana* LONGIFOLIA homologous proteins	grain length and width	(Wang SK. et al., 2015; Wang YX. et al., 2015)
	*GW8*	squamosa promoter binding protein-like	grain length and width	(Wang et al., 2012)
	*AH2*	MYB family transcription factor	grain size	(Ren et al., 2019a)

### Regulatory network between rice grain type and signaling pathways

The latest reports show that the grain type of rice is regulated by signaling pathways such as BR, G-protein, and ubiquitin-proteasome (Li N. et al., 2019; Ren et al., 2023). Hu et al. successfully isolated and cloned an important gene, *Grain Size2* (*GS2*), from a local Zhejiang rice variety, “Baodali”, which can significantly improve rice yield. The GS2 protein is localized in the nucleus and regulates the length and width of grains by promoting cell division and expansion (Hu et al., 2015). GS2 directly interacts with the negative regulator of the BR signal transduction pathway, and its transcriptional activation activity is inhibited. In addition, it was found that *Grain Length2* (*GL2*) and *GS2* were located at the same locus. The *GL2* gene increased the grain size, and significantly increased the number of tillers and grains per ear, thus increasing the yield per plant (Che et al., 2015). Additionally, the gene *Oryza sativa MicroRNA396* (*OsmiR396*) can cleave *GS2*, but the rare AA allele variation of *GS2* will affect the binding site of *OsmiR396*, resulting in its inability to cleave *GS2*, thus increasing the expression of *GS2.* This indicates that the AA allelic variation of *GS2* can increase grain size (Duan et al., 2015). These studies point to a new strategy that can be used to generate new high-yielding rice cultivars. Gγ subunits in rice, including G PROTEIN GAMMA SUBUNIT3 (GS3), G PROTEIN GAMMA SUBUNIT2 (GGC2), and DENSE PANICLE 1 (DEP1), are involved in the regulation of grain type. Among them, *GS3* combines Gβ protein by competing with *GGC2* and *DEP1* genes to reduce grain length, while *GGC2* and *DEP1* genes are associated with Gβ protein interaction to increase grain length (Sun et al., 2018). In previous studies, the dominant gene at the *DEP1* gene locus was caused by acquired mutations, which can promote cell division, increase the number of branches and grains per panicle, and thus promote an increase in rice yield. This gene has been widely used in rice yield-increasing varieties in China (Huang et al., 2009). Song et al. discovered a gene called *Grain Width2* (*GW2*) that influences rice grain width and weight. This gene encodes an E3 ubiquitin ligase and is involved in the regulation of the ubiquitin-proteasome signaling pathway. Without functional *GW2*, the substrate that should undergo degradation is not specifically identified, leading to an increased cell number and a quicker filling rate. Consequently, this broadens the grain shape and enhances the grain weight of rice, ultimately boosting the overall yield of the rice crop (Song et al., 2007). Recent studies have found that the *GW2*-*Wide Grain1* (*WG1*)-*Oryza sativa Basic Leucine Zipper47* (*OsbZIP47*) pathway regulates rice seed size. *WG1* encodes glutaredoxin, which controls seed size by promoting cell proliferation, while *OsbZIP47* limits seed growth by reducing cell proliferation. Further research and analysis showed that *GW2* can ubiquitinate *WG1* and degrade it. This study provides a new target for understanding the mechanism of rice grain development and has an important reference value for improving rice yield (Hao et al., 2021). Additionally, some laboratories have shown that the MAPK cascade signaling pathway is an essential regulator of seed size and has potential value for improving crop yield. Xu et al. identified a *grain size and number1 (gsn1)* mutant, *GSN1*, which encodes the mitogen-activated protein kinase phosphatase *Oryza sativa* MAPK PHOSPHATASE1 (OsMKP1). When OsMKP1 loss-of-function results in larger but fewer grains, its overexpression produces smaller but more grains. The additional analysis documented that OsMKP1 interacts with the *Oryza sativa* MAPK PHOSPHATASE6 (OsMAPK6), leading to its dephosphorylation and inactivation. Further research found that OsMKP1 can also inhibit *Oryza sativa* MAPK PHOSPHATASE10 (OsMKKK10) and *Oryza sativa* MAPK PHOSPHATASE4 (OsMKK4*)* (Guo et al., 2018; Xu et al., 2018a; Xu et al., 2018b). Thus, these studies revealed that *GSN1* determines grain size by inhibiting the MAPK signaling pathway and establishing the *GSN1*-MAPK module; it laid the foundation for revealing the mechanism of rice grain size regulation.

### Regulatory network for rice grain type and quality

The yield and quality of rice cannot be achieved simultaneously, so balancing the relationship between the two to obtain high-yielding, high-quality varieties is a big challenge. *Grain Length7* (*GL7*) is the major rice gene regulating grain length and width. The *GL7* gene, which is responsible for controlling the shape and quality of rice grains, has been successfully isolated. By modifying the pattern of cell division, *GL7* participates in regulating the longitudinal elongation of cells. Further meticulous genetic analysis demonstrated that a DNA fragment containing 17.1 kb of the *GL7* locus in American long-grain rice varieties underwent tandem duplication, which increased the expression of the *GL7* gene but also caused the down-regulation of the expression of its adjacent negative regulator. The above two conditions are jointly regulated to cause an increase in grain length, a decrease in chalk content, and chalkiness. These changes improve the appearance and taste of rice (Wang SK. et al., 2015; Wang YX. et al., 2015). It has been documented that an allelic variation of the *Grain Width8* (*GW8*) gene in high-quality Pakistani Basmati rice, which changes the grain morphology to be more elongated, significantly improves rice quality but causes a 14% loss in rice yield (Wang et al., 2012). *Grain Width7* (*GW7*) and *GL7* are located at the same genetic locus. Further studies revealed that *GW8* binds directly to the promoter of the *GW7* gene and inhibits its expression. The *GW7* gene undergoes allelic variation and becomes a semi-dominant allele *GW7^TEA^*, which can make its gene expression level not regulated by *GW8*. Therefore, controlling the *GW8*-*GW7* module can significantly improve rice quality by ensuring that the yield does not decrease. The results also showed that applying optimal allelic variants of the *GW7* and *GS3* genes to indica rice, which produces a high yield in China, could significantly improve rice quality and yield (Wang SK. et al., 2015). The above study identified new genes with important implications for molecular module design and breeding of high-yielding and high-quality rice. It also provides a new clue to unraveling the molecular nature of synergistic genetic modification of rice quality and yield. In a news report in *Nature* on the results of this study, Professor Susan McCouch of Cornell University was quoted as saying that no one had taken into account both high yield and high quality in rice breeding, “which will be **[SIC]** a huge impact on research results. “ Academician Qian Qian’s team at the China Rice Research Institute also recently published a paper to discuss precise and efficient strategies for improving rice grain size and rice quality, with the aim of obtaining a germplasm resource bank of excellent seed diameter and grain quality genes and achieving both high yield and high quality of rice (Ren et al., 2023). Glumelles and lemmas are unique organs of graminoids that also determine grain size and quality. Recent studies have shown that *Abnormal Hull2* (*AH2*) encodes an MYB domain protein and functions during glume and grain development. The grain of *AH2* mutants is smaller, and its quality is changed due to decreased amylose content, gel consistency, and increased protein content. At the same time, a part of the glumes loses its outer silicified cells, resulting in a conversion from the outer rough epidermis to the inner smooth epidermal cells (Ren et al., 2019a). The analysis of the *AH2* function helps to understand the potential mechanisms regulating rice grain shape and is important for improving rice yield and quality.

## Research advances in molecular design breeding

**Figure 2:**
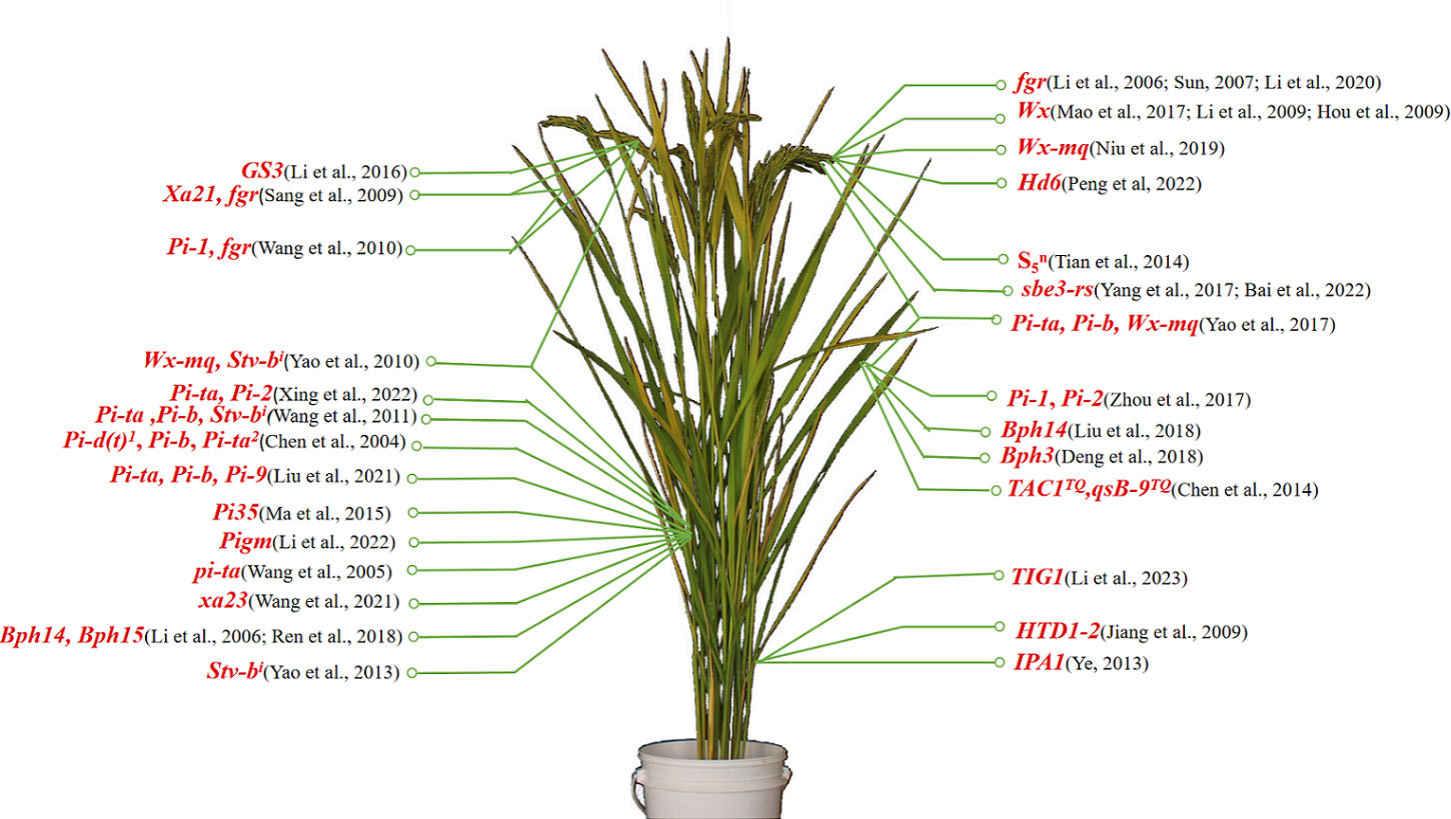
Distribution map of functional gene molecular markers developed in China.

Striking a balance between yield and quality in rice - much like the proverbial challenge of having both fish and bear’s paws - is an intricate task but critical for future global food security. The field of rice breeding is constantly evolving, fueled by rapid advances in molecular biology, genomics, and bioinformatics. The techniques and methods used in rice breeding have undergone continuous refinement and innovation, ranging from traditional crossbreeding and mutation breeding to more modern approaches such as molecular marker-assisted breeding molecular design breeding, and gene editing breeding. These technologies can combine different rice yield traits to produce high-yielding, high-quality varieties. At the same time, they can also combine various excellent characteristics, such as disease resistance, drought resistance, lodging resistance, and high-quality grain, leading to the cultivation of superior rice varieties and promoting sustainable agriculture. Many functional genes have been identified, and scientists have employed some of them to develop corresponding molecular markers (**Table 4**; **Figure 2**). This has resulted in the cultivation of numerous superior rice varieties, significantly boosting the efficiency of rice breeding and enabling the breeding of rice varieties that incorporate numerous advantages and benefits.

**Table 4:** Functional gene molecular markers developed in China.

Donor parent	Recipient parent	Gene	Reference
Yuefeng B	320B without fragrance	*fgr*	(Li JH. et al., 2006)
Chuanxiang 29B	lemont, R2, Mei B, P18, G46B	*fgr*	(Sun, 2007)
Daohuaxiang 2	Kongyu 131	*fgr*	(Li et al., 2020)
Xiangjing 9407	IRBB60	*Xa21, fgr*	(Sang et al., 2009)
BL122, XH74	R290	*Pi-1, fgr*	(Wang et al., 2010)
CBB23	Chuanhui 907, Chuanhanghui 908, Chuanhui 991, Chuanhui 992	*xa23*	(Wang et al., 2021)
Mowanggu, Akihikari	IR64, Teqing	*Wx*	(Mao et al., 2017)
K81	Chuanxiang 29B, IR58025B, BoIIIB	*Wx*	(Li et al., 2009)
HC086	R838, R898, R476, R6547	*Wx*	(Hou et al., 2009)
Nanjing 9108	Shen 01B	*Wx-mq*	(Niu et al., 2019)
Kantou 194	Wuyujing 7	*Stv-b^i^*	(Yao et al., 2013)
Kantou 194	Wuyujing 7	*Wx-mq, Stv-b^i^*	(Yao et al., 2010)
Jiangtangdao 1	Miyang 23	*sbe3-rs*	(Yang et al., 2012)
Jiangtangdao 1	Hudao 89	*sbe3-rs*	(Bai et al., 2023)
Sanlicun, IAC1246, JAPPENI TUNGKUNGO, MIGA	SAGC-4	*GS3*	(Li et al., 2016)
htd1-2, Taibei 309	htd1-2, 9311	*HTD1-2*	(Jiang et al., 2009)
TN1, TN8, TN19, TN21, TN28, rice-1, rice-2, E6, ipa1-2010	Changhui 891, Changhui 121, Changhui T025, Changhui T470 and so on	*IPA1*	(Ye, 2013)
Zhendao 88	Wulingjing 1	*TAC1^TQ^, qsB-9^TQ^*	(Chen et al., 2014)
Fukei 138	LTH, Nipponbare, Kenjiandao 3, Kenjiandao 6, Kendao 8, Kendao 12, Kendao 16, Suijing 3, Longjing 10, Longhua00-835, Longjing 13, Longjing 34	*Pi35*	(Ma et al., 2015)
Kongyu 131	Zhennuo 19	*Pigm*	(Li et al., 2022)
RBR1-2	Guangzhan 63S	*Pi-1, Pi-2*	(Zhou GL. et al., 2017)
Katy	RU9101001	*Pi-ta*	(Wang et al., 2005)
Digu, BL-1, Pi-4	G46B, Jiangnanxiangnuo	*Pi-d(t)^1^, Pi-b, Pi-ta^2^*	(Chen et al., 2004)
Wuyunjing 8	Yandao 10	*Pi-ta, Pi-b, Pi-9*	(Liu et al., 2021)
Nanjing 5055	Wujing 15	*Pi-ta, Pi-b, Wx-mq*	(Yao et al., 2017)
Wuyunjing 8	Zhendao 42	*Pi-ta, Pi-b, Stv-b^i^*	(Wang J. et al., 2011)
H401	Lianjing 05-45	*Pi-ta, Pi-2*	(Xing et al., 2023)
Nipponbare, 9311, Kantou 194, Ning 9108, Nanjing 11, Bijing 43.Bijing 44, Bijing 45	02428, Peiai 64S	*S_5_^n^*	(Tian et al., 2014)
O. rufipogon Griff.	Zhenshan 97	*TIG1*	(Li et al., 2023)
Kasalath, Shuhui 498	Nipponbare, Suxiu 867	*Hd6*	(Peng et al., 2022)
B5	Huhan 1B, Xiushui 123, Huhan 7B, Huanghuazhan, Zhongzu 14, Huhan 15, Nipponbare	*Bph14*	(Liu et al., 2018)
Rathu Heenati	9311	*Bph3*	(Deng et al., 2018)
B5	9311, 1826	*Bph14, Bph15*	(Li JB. et al., 2006)
B5	C815S	*Bph14, Bph15*	(Ren XM. et al., 2018)

## Advances in key technologies for molecular design breeding

### CRISPR/Cas9 technology for gene editing and breeding

Rice genome editing technology refers to the precise modification of genes in rice to improve its characteristics (Voytas, 2013), such as increasing plant resistance to disease, stress, and yield, making it possible to cultivate excellent rice varieties. CRISPR/Cas9 genome editing technology has been widely used for rice fertility and to improve resistance, quality, and agronomic traits. It has developed into a mainstream gene editing system, which is important for future rice breeding. In recent years, many scientists have used CRISPR/Cas9 technology to perform genome editing and have obtained many high-quality rice germplasm resources. Song et al. used this technology to edit the ideal plant type gene, *IPA1*, and obtained a material, IPA1-Pro10, which can increase both the number of tillers and the number of grains per panicle, thus greatly promoting rice yield (Song et al., 2022). In terms of rice quality improvement, Huang et al. used this technology to edit the promoter of the *Waxy* (*Wx*) gene, which controls the amylose content (AC) in rice grains, and create novel *Wx* alleles with fine AC levels, improving eating and cooking quality (ECQ) (Huang et al., 2020). This technology balances beneficial complementary traits and greatly facilitates the process of cultivating high-quality germplasm.

### Apomixis

The utilization of heterosis has markedly improved rice yield, but it is hampered by trait separation in hybrid offspring, and heterosis cannot be maintained. Based on this, the possibility has been advanced to transform the process of sexual reproduction of rice to apomictic reproduction so that hybrid offspring would have the same genotype as the parents and heterosis would be preserved. Apomixis is a plant that produces a clone of itself through asexual reproduction by seed, and this strategy could be revolutionary for agriculture (Liu et al., 2023). BABY BOOM (BBM), a member of the AP2 family of transcription factors specifically expressed in spermatids, continues to be expressed in zygotes after fertilization. Thus, this transcription factor may be necessary during the initiation of embryonic development (Horstman et al., 2014). Sundaresan’s research group demonstrated that *BBM1* may play a crucial role in zygotic embryos and that ectopic expression of *BBM1* allows female cells to bypass fertilization and form normal embryos through parthenogenesis. By editing three *MiMe* genes and ectopically expressing the *BBM1* gene, the replacement of meiosis by mitosis was achieved, establishing an apomictic reproductive system in rice and allowing the asexual reproduction of rice seeds (Khanday et al., 2018). During the same period, the study performed by Kejian Wang’s team at the China National Rice Research Institute showed that the heterozygosity of F_1_ hybrid rice could be fixed. These researchers employed the Chunyou 84 hybrid rice cultivar as a parent to edit three genes using CRISPR/Cas9 technology, *PAIR1*, *OsREC8*, and *OsOSD1*, which are involved in the meiotic phase of rice, and the *MATRILINEAL* (*MTL*) gene, which is homologous to the maize haploid induction gene. The resulting tetraploid mutant rice replaced meiosis with a division process similar to mitosis, generating progeny with a genotype identical to that of the parents and achieving the fixation of heterozygous genotypes (Wang et al., 2019). Although both methods realize apomictic reproduction in rice, they affect rice fertility to a certain degree. Therefore, future research should focus on the improvement of apomictic reproduction to realize its application in rice farming.

## Molecular design breeding for rice variety improvement

**Figure 3:**
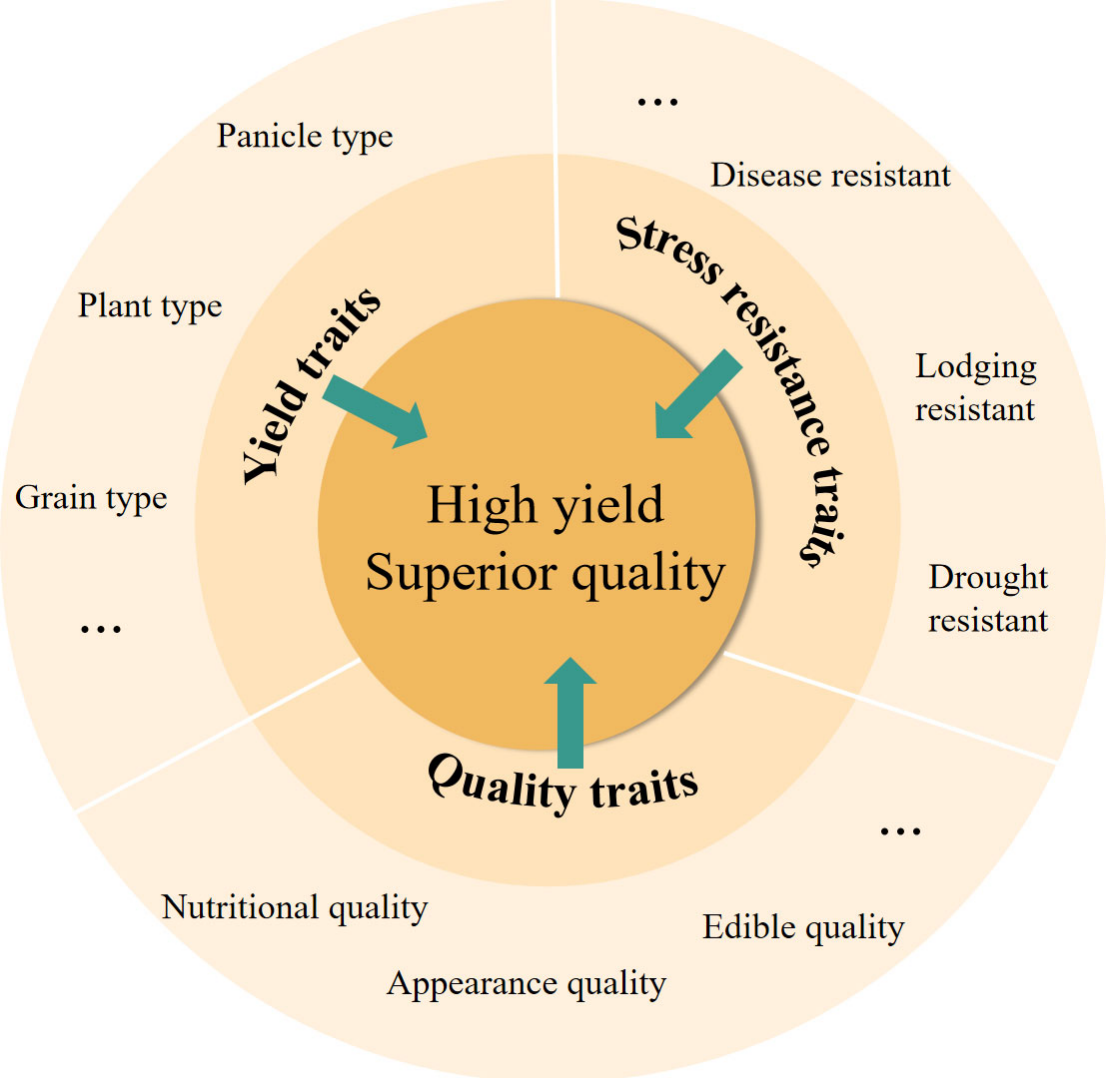
Reasonable molecular design breeding strategy.

Molecular design breeding is a technology that uses gene editing, synthetic biology, and other modalities to directly manipulate DNA sequences. This approach expedites the breeding process and enhances its efficiency (Wang JK. et al., 2011). The rational aggregation of high-quality genes through molecular design methods is conducive to the breeding of superior new rice varieties (**Figure 3**). This technology has allowed researchers to consolidate numerous genes for breeding, marking a significant milestone in the history of rice breeding. Utilizing the established gene regulatory network for yield traits to conduct molecular design breeding holds great promise for enhancing crop yield and quality. High-quality genes from Japonica Nipponbare and Indica 9311 were used as desirable target gene donors to design 28 genes affecting rice yield, appearance quality, culinary quality, and ecological adaptability. With its inferior culinary quality, the ultra-high-yield cultivar “Teqing” served as a recipient of these desirable genes. After extensive periods of hybridization, backcrossing, and polymer selection, combined with directional selection using molecular markers, several exceptional offspring materials were produced. These offspring materials fully retained the genetic background and high-yielding characteristics of Teqing, while significantly improving the visual quality, culinary quality, taste, and flavor of the rice. The quality of the corresponding hybrid rice was also markedly enhanced (Zeng et al., 2017a). These findings embody an excellent transition from traditional crop breeding to efficient, precise, and directed molecular design breeding, and they are likely to inspire similar future efforts.

A significant research effort led to the construction of a pan-genomic dataset of the *O.sativa-O.rufipogon* complex, which was generated by deep sequencing and reassembly of 66 different cultivars. The genome comparison identified 23 million sequence variants in the rice genome. These sequence variants, including many known quantitative traits, will help identify novel causal variants that constitute complex traits. Most importantly, this pan-genomic dataset was used to analyze the entire set of coding genes, revealing many variants in different rice cultivars (Zhao et al., 2018). This pan-genomic resource will promote evolutionary and functional studies of plant architecture and grain development in rice, and provide a reference for rice design and breeding. In addition, the polygenic (*GHD10*-*GS2*-*DEP1*-*IPA1*) pyramiding effect was studied, and a pyramiding breeding model for high-yielding genes was designed using the cloned series of yield-affecting genes (Zeng et al., 2017b). The model elaborates the molecular breeding theory based on the utilization of heterosis between the indica and japonica subspecies and provides a theoretical basis for the future design of super hybrid rice, offering a feasible strategy to achieve the third leap in rice production yield.

The “Zhongke 804” and “Zhongkefa” series are landmark new rice cultivars developed by Jiayang Li, a member of the Chinese Academy of Sciences and a researcher at the Institute of Genetics and Developmental Biology of the Chinese Academy of Sciences. These cultivars were developed based on the theoretical foundation and design concept of “Molecular Mechanism and Cultivar Design of Rice High-yield and High-quality Traits,” and represent important achievements made with the support of the Class A Strategic Pilot Science and Technology Special Project “Innovation Systems of Molecular Module Design Breeding” of the Chinese Academy of Sciences. Compared with the main high-quality rice cultivar, “Daohuaxiang, “ farmed in Northeast China, the new japonica rice cultivars “Zhongke 804” and “Zhongkefa” series have the characteristics of high quality, high yield, blast resistance, lodging resistance, and better taste (Zeng et al., 2017a). Given the characteristics and production needs of the northeastern region of rice farming, the research group of Jiayang Li, in cooperation with the teams of Qian at the China National Rice Research Institute and Guomin Zhang at the Northern Japonica Rice Molecular Breeding Joint Research Center, have developed new varieties, “Zhongkefa 5” and “Zhongkefa 6”, which are characterized by high yield, high quality, and strong stress resistance (GUO et al., 2019). In addition, Jiayang Li’s research group also developed a new conventional early maturing soft japonica rice variety, “Zhongkefa 928,” based on excellent quality-related genes and blast resistance genes through in-depth analysis and molecular design, using new molecular design breeding techniques (Yu et al., 2018). As a typical example of the transition from scientific theory to production practice, these varieties will provide a higher level of sophistication to the field of variety design and breeding research and greatly promote the transition from traditional crop breeding to efficient breeding based on a precise and targeted molecular design.

Due to the pleiotropy between genes, potential genetic instability, and complex regulatory networks among genes, few gene resources are available for improving yield traits in rice varieties. When using molecular design techniques to aggregate excellent genes, it is also difficult to balance the expression of each gene. Therefore, further exploring the genes of excellent yield traits and revealing the regulatory networks and genetic mechanisms between each gene is significant for cultivating high-yielding and high-quality varieties and establishing excellent germplasm.

## Prospects

In recent years, the challenge of ensuring national food security through the cultivation of high-yielding, high-quality rice varieties has been exacerbated by population growth and shrinking agricultural land. Chinese scientists have accomplished numerous significant breakthroughs in rice yield trait research. They have applied advanced breeding techniques like molecular markers and molecular design, cloned an array of rice yield trait genes, and amassed a wealth of excellent genes to breed several high-quality rice varieties. For instance, they have used CRISPR/Cas9 and apomixis technology for germplasm improvement and used molecular design breeding methods to cultivate superior rice varieties, such as the Zhongkefa series.

Based on the existing functional genes and regulatory networks, further mining of related genes and analysis of gene regulatory networks, developing and using molecular markers with application value, the establishment of a high-throughput and low-cost genotype detection system through multiple gene pyramiding, gene editing, and apomixis, the creation of excellent new breeding materials, and cultivating new rice varieties with high yield and quality will be an inevitable trend in future rice research.

## Author contributions

YR, YM and YW conceived and designed the article framework. QZ wrote the manuscript. QZ, YR, QJ, WY and YW revised the manuscript. All authors contributed to the article and approved the submitted version.
